# Paricalcitol Pretreatment Attenuates Renal Ischemia-Reperfusion Injury via Prostaglandin E_2_ Receptor EP4 Pathway

**DOI:** 10.1155/2017/5031926

**Published:** 2017-03-29

**Authors:** Yu Ah Hong, Keum Jin Yang, So Young Jung, Ki Cheol Park, Hyunsu Choi, Jeong Min Oh, Sang Ju Lee, Yoon Kyung Chang, Cheol Whee Park, Chul Woo Yang, Suk Young Kim, Hyeon Seok Hwang

**Affiliations:** ^1^Division of Nephrology, Department of Internal Medicine, College of Medicine, The Catholic University of Korea, Seoul, Republic of Korea; ^2^Clinical Research Institute, Daejeon St. Mary's Hospital, Daejeon, Republic of Korea

## Abstract

The protective mechanism of paricalcitol remains unclear in renal ischemia-reperfusion (IR) injury. We investigated the renoprotective effects of paricalcitol in IR injury through the prostaglandin E_2_ (PGE_2_) receptor EP4. Paricalcitol was injected into IR-exposed HK-2 cells and mice subjected to bilateral kidney ischemia for 23 min and reperfusion for 24 hr. Paricalcitol prevented IR-induced cell death and EP4 antagonist cotreatment offset these protective effects. Paricalcitol increased phosphorylation of Akt and cyclic AMP responsive element binding protein (CREB) and suppressed nuclear factor-*κ*B (NF-*κ*B) in IR-exposed cells and cotreatment of EP4 antagonist or EP4 small interfering RNA blunted these signals. In vivo studies showed that paricalcitol improved renal dysfunction and tubular necrosis after IR injury and cotreatment with EP4 antagonist inhibited the protective effects of paricalcitol. Phosphorylation of Akt was increased and nuclear translocation of p65 NF-*κ*B was decreased in paricalcitol-treated mice with IR injury, which was reversed by EP4 blockade. Paricalcitol decreased oxidative stress and apoptosis in renal IR injury. Paricalcitol also attenuated the infiltration of inflammatory cells and production of proinflammatory cytokines after IR injury. EP4 antagonist abolished these antioxidant, anti-inflammatory, and antiapoptotic effects. The EP4 plays a pivotal role in the protective effects of paricalcitol in renal IR injury.

## 1. Introduction

Renal ischemia-reperfusion (IR) injury is a major cause of acute kidney injury (AKI) [1, 2]. Oxidative stress and inflammation are known to play an important role in the parenchymal damage of IR injury [3]. Production of reactive oxygen species is increased after IR injury and it has been demonstrated that oxidative stress induced inflammatory cells infiltration into the renal interstitium and release of proinflammatory cytokines [4–6]. These pathologic processes lead to apoptotic cell death and inflammatory cascades also exacerbate oxidative stress around the inflamed lesion [7–9]. Therefore, inhibition of oxidative stress, renal inflammation, and apoptosis is an important approach to ameliorate ischemic kidney injury.

A large number of studies have shown that vitamin D and its analogs protect against kidney damage by reducing oxidative stress, inflammation, and apoptosis in experimental models, such as diabetic nephropathy and unilateral ureteral obstruction [10, 11]. We previously demonstrated that paricalcitol (19-nor-1, 25-dihydroxyvitamin D_2_) effectively inhibits inflammation in renal IR injury via the upregulation of cyclooxygenase-2 (COX-2) and prostaglandin E_2_ (PGE_2_) [12]. Biological functions of PGE_2_ rely on the receptor subtypes EP1 to EP4, which activate diverse signaling pathways through coupling to different G proteins [13]. In particular, EP4 signaling activates the phosphatidylinositol 3-kinase (PI3K) pathway leading to Akt activation [14, 15] and modulates the immune response in myocardial and cerebral ischemia models [16, 17]. However, it remains unclear whether paricalcitol pretreatment attenuates oxidative stress, inflammation, and apoptosis in renal IR injury via the PGE_2_-EP4 signaling pathway.

Therefore, we investigated the role of PGE_2_-EP4 signaling in paricalcitol effect on renal IR injury. This study aimed to determine whether paricalcitol modulates the PGE_2_-EP4 signaling and the EP4-dependent pathway is critical for the renoprotective effects of paricalcitol through preventing oxidative stress, inflammation, and apoptosis after renal IR injury.

## 2. Materials and Methods

### 2.1. Drugs and Animal Model of Renal IR Injury

Seven- to eight-week-old male C57BL/6J mice, weighing 22 to 23 g, were divided into seven groups and housed under a 12 h light-dark cycle. Food and water were freely available. Paricalcitol (0.3 *μ*g/kg, Abbott Laboratories, IL, USA) and AH-23848 (2 mg/kg, Cayman Chemical, MI, USA), an EP4 antagonist, were injected intraperitoneally 24 h before ischemia [11, 12, 18]. AH-23848 was dissolved in 10% NaHCO_3_ solution and then diluted with saline to give the same volume as paricalcitol. The sham and IR groups of mice received an equivalent volume of saline.

The renal IR injury was performed under anesthesia by intraperitoneal injection of a mixture of tiletamine-zolazepam (30 mg/kg) and xylazine (10 mg/kg). Under anesthesia, the kidneys were exposed by a midline incision and the bilateral renal pedicles were occluded for 23 min using nontraumatic microaneurysm clamps and core body temperature was maintained using a homoeothermic pad. After clamp removal, the abdomen was closed. Sham-operated mice received an identical surgical procedure, except for the occlusion of renal pedicles [12, 19, 20]. The mice were sacrificed under same anesthesia and tissue specimens and blood samples were collected at 24 h reperfusion. The kidneys were harvested for analysis of Akt, pS473 Akt, and p65 NF-*κ*B at 6 h of reperfusion. The animal experiments ethics committee of our institution approved the experimental protocol and the experiments were performed in accordance with our institutional animal care guidelines.

### 2.2. Quantification of Functional and Morphological Changes in Kidney Injury

Serum creatinine concentrations were measured using an IDEXX VetTest® Chemistry Analyzer (IDEXX Laboratories, Inc., ME, USA). The kidney tissues were embedded in paraffin after fixing with formalin buffer and cut into 3 *μ*m thick sections. A tubular necrosis grading scale of 0–5 from hematoxylin and eosin staining, as outlined in a previous report, was used to assess the degree of tubular injury [12]. All of these sections were examined in a blinded manner using light microscopy.

### 2.3. Immunohistochemistry and Immunofluorescence Staining

We detected apoptosis by the terminal deoxynucleotidyl transferase-mediated dUTP nick end-labeling (TUNEL) using the ApopTag Plus Peroxidase In Situ Apoptosis Kit (Millipore, MA, USA). Kidney sections were prepared according to the procedure of the Vectastain Elite ABC kit (Vector Laboratory, CA, USA). Anti-CD3 antibody (Santa Cruz Biotechnology, CA, USA) and anti-F4/80 antibody (Abcam, Cambridge, UK) were used and revealing reaction was performed with an AEC Chromogen Kit (Immunotech, Marseille, France). All of these sections were examined in a blinded manner using light microscopy. The number of positive cells was quantified per high power field for each kidney and at least 20 fields were reviewed for each slide.

For immunofluorescence staining, sections were incubated with anti-EP4 antibody (Santa Cruz Biotechnology) and anti-aquaporin-1 (AQP-1) antibody (Millipore) overnight and then visualized with anti-rabbit Alexa Fluor-488 and -594 (Life Technologies, OR, USA). The images were captured by confocal microscopy (LSM5 Live Configuration Variotwo VRGB; Zeiss, Germany).

### 2.4. Cultured Human Proximal Tubular Cell Experiments

Human proximal tubular epithelial (HK-2) cells (American Type Culture Collection, VA, USA) were grown in Dulbecco's modified Eagle's medium (DMEM) supplemented with 10% fetal bovine serum, 50 U/ml penicillin, and 50 *μ*g/ml streptomycin. When the cells reached 80% confluence, the culture medium was replaced with serum-free DMEM for 16 h. Cells were harvested after treatment with 0.2, 2.0, and 20 ng/ml paricalcitol for 24 h to assess the effect on expression of vitamin D receptor (VDR), PGE_2_ synthetic enzymes, and PGE_2_ receptors.

HK-2 cells were pretreated with paricalcitol (2 ng/ml) or AH-23848 for 1 h and then exposed to ischemia by immersing the cellular monolayer in mineral oil (Sigma-Aldrich, MO, USA) for 90 min [21]. AH-23848 was dissolved in NaHCO_3_ and diluted with distilled water. The cells were harvested for analysis of cyclic AMP responsive element binding protein (CREB) and Akt expression at 15 min and 30 min reperfusion. The cells were also harvested for analysis of p65 NF-*κ*B phosphorylation at 15 min reperfusion.

### 2.5. Measurement of Cell Viability

Cell viability was determined using a commercially available 3-(4,5-dimethylthiazol-2-yl)-2,5-diphenyltetrazolium bromide (MTT) assay (EZ-Cytox; Daeil Lab Service, Korea) after 24 h of IR. The optical densities of the samples were determined at 450 nm in a microplate reader (Bio-Rad Laboratories, CA, USA).

### 2.6. Immunoblot Analyses of HK-2 Cells and Kidney Tissue

We performed immunoblot analyses for mice kidneys and HK-2 cell lysates as described previously [12]. The proteins were separated by SDS-polyacrylamide gel electrophoresis, transferred to nitrocellulose membranes, and incubated with antibodies against the following proteins: VDR (Santa Cruz Biotechnology); COX-2, Akt, pS473 Akt, CREB, pS133 CREB, pp65 NF-*κ*B, BCL-2-associated X (Bax), B-cell leukemia/lymphoma 2 (Bcl-2), and glyceraldehyde-3-phosphate dehydrogenase (GAPDH) (Cell Signaling Technology, MA, USA); EP4, p65 NF-*κ*B, superoxide dismutase (SOD) 1 and SOD2 (Abcam); *β*-actin (Sigma-Aldrich). The membranes were then incubated with horseradish peroxidase-conjugated anti-rabbit IgG or anti-mouse IgG (Invitrogen Biotechnology, CA, USA) and positive bands were detected and analyzed using the ChemiDoc XRS Image system (Bio-Rad Laboratory).

### 2.7. Nuclear and Cytoplasmic Fractionation of Kidney Tissue

Nuclear and cytoplasmic fractionation of kidney tissues was performed using a Nuclear/Cytosol Fractionation kit (BioVision Inc., CA, USA) after single cellularization with 1 ml of 1 mg/ml collagenase. After treatment with CEB-A and CEB-B, the cytoplasmic extract was harvested by centrifugation at 16,000*g* for 5 min. The pellet was resuspended in 100 *μ*l of NEB mix and the nuclear fraction was harvested by centrifugation at 16,000*g* for 10 min.

### 2.8. Fractionation of Cellular Membrane and Cytoplasm of HK-2 Cells

Membrane and cytoplasmic fractionation of HK-2 cells was performed using a Mem-PER Plus Membrane Protein Extraction Kit (Thermo Scientific, IL, USA). Briefly, the cells were washed with Cell Wash Solution, resuspended in permeabilization buffer, and incubated for 10 min with constant mixing at 4°C. The cytoplasmic supernatant was harvested after centrifugation at 16,000*g* for 15 min. The pellet was resuspended in solubilization buffer and the solubilized membrane fraction was harvested after centrifugation for 30 min at 4°C.

### 2.9. Enzyme Immunoassay

HK-2 cells and kidney tissues were homogenized in PRO-PREP™ Protein Extraction Solution (iNtRON Biotechnology, Korea) and then centrifuged for 10 min at 12,000 rpm. Equal amounts of protein were assayed by PGE_2_ enzyme-linked immunosorbent assay (ELISA) (Cayman Chemical) and thromboxane B_2_ (TXB_2_) ELISA kit (Enzo Life Science, NY, USA). The optical densities of the samples were determined at 450 nm in a microplate reader (Bio-Rad Laboratory).

### 2.10. Small Interfering RNA

Small interfering (si) RNAs targeted to EP4 (Bioneer, Daejeon, Korea) and scrambled siRNA (siRNA control, Bioneer) were complexed with transfection reagent (Invitrogen Biotechnology) according to the manufacturer's instructions. HK-2 cells were transfected with EP4 siRNAs for 24 h by transfection reagent (G-Fectin) according to the manufacturer's instructions. Twenty-four hours after transfection, cells were treated with paricalcitol 1 h before ischemia. The cells were harvested for analysis of CREB and Akt expression at 15 min and 30 min reperfusion. The cells were also harvested for analysis of p65 NF-*κ*B phosphorylation at 15 min reperfusion.

### 2.11. Immunoprecipitation of EP4

Kidney tissues were lysed with kinase buffer (KB; 50 mM Tris-HCl (pH 7.4), 25 mM NaF, 40 mM *β*-glycerol phosphate (pH 7.4), 120 mM NaCl, and 1% NP40) and then 1 mg of lysate immunoprecipitated using 1 *µ*g of anti-EP4 antibody (Santa Cruz Biotechnology) and protein G Sepharose 4 Fast Flow (GE Healthcare, Sweden). After washing with KB without 1% NO40, immunoblotting was performed using by PGE_2_ antibody (Biorbyt Ltd., UK).

### 2.12. Assessment of Markers for Renal Oxidative Stress

Lipid peroxidation as an index of oxidative stress was determined by assaying malondialdehyde (MDA) production with the thiobarbituric Acid Reactive Substance (TBARS) test (OxiSelect MDA Adduct ELISA Kit, Cell Biolabs Inc., CA, USA) in kidney tissue after IR injury pretreated with paricalcitol and/or AH-23848 according to the manufacturer's protocol.

### 2.13. Real-Time Reverse Transcription PCR of HK-2 Cells and Kidney Tissue

Total RNA was isolated from kidney tissues and HK-2 cells using a NucleoSpin® RNA II kit (Macherey-Nagel, Germany). cDNA was synthesized using Reverse Transcriptase Premix (Elpis Biotech, Korea) and amplified in a Power SYBR® Green polymerase chain reaction (PCR) Master Mix (Applied Biosystems, CA, USA) with gene-specific primer pairs (Table 1). Quantitative real-time PCR was performed on an ABI 7500 FAST instrument (Applied Biosystems). The expression levels of mRNAs were normalized to expression of* GAPDH*.

### 2.14. Statistical Analysis

Data are expressed as the mean ± SEM. Multiple comparisons were performed using one-way analysis of variance with Tukey's post hoc test using SPSS 20.0 software (IBM, NY, USA). A *P* < 0.05 was considered statistically significant.

## 3. Results

### 3.1. Paricalcitol Upregulates COX-2 and PGE_2_ Expression in HK-2 Cells

**Figure 1:**
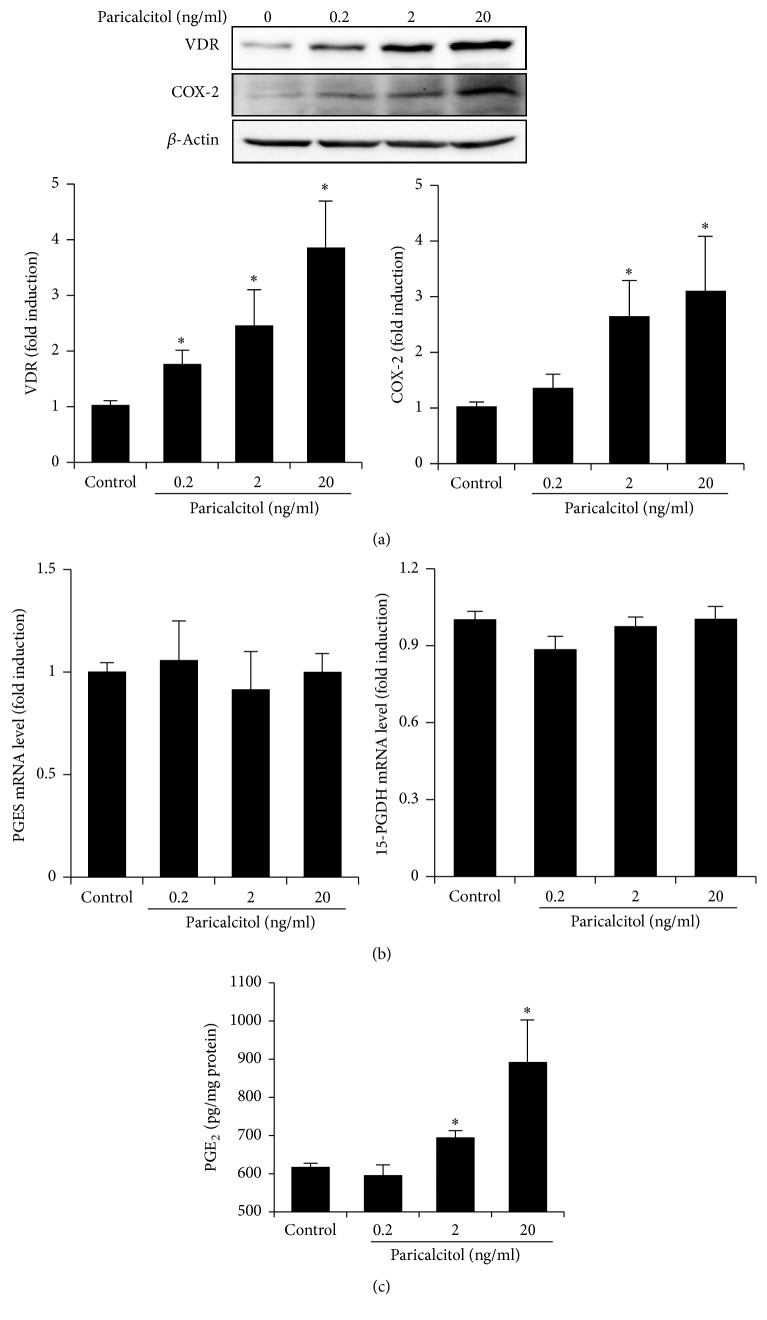
Paricalcitol upregulated the COX-2 and PGE_2_ pathway in HK-2 cells. (a) Semiquantitative immunoblotting revealed that paricalcitol significantly increased VDR and COX-2 expression in a dose-dependent manner. (b) RT-PCR results indicated that paricalcitol treatment did not affect expression of* 15-PGDH* and* PGES*. (c) The PGE_2_ level was significantly elevated in paricalcitol-treated cells compared to control cells. Each column represents the mean ± SEM of three independent experiments. ^*∗*^*P* < 0.05 versus control.

Figure 1 shows the effects of paricalcitol on VDR, PGE_2_, and its synthetic and catalytic enzymes in HK-2 cells. Expression of VDR and COX-2 increased significantly after paricalcitol treatment in a dose-dependent manner (Figure 1(a)). However, paricalcitol did not affect the mRNA levels of* prostaglandin E synthases (PGES)*, the enzyme that isomerizes the PGH_2_ to PGE_2_, or* 15-hydroxyprostaglandin dehydrogenase (15-PGDH)*, the enzyme that catalyzes conversion of PGE_2_ to a less bioactive form (Figure 1(b)). HK-2 cells produced a significantly higher level of PGE_2_ than control cells after treatment with 2 and 20 ng/ml of paricalcitol (Figure 1(c)).

### 3.2. Paricalcitol Upregulates Cellular Membrane Expression of EP4 in HK-2 Cells

**Figure 2:**
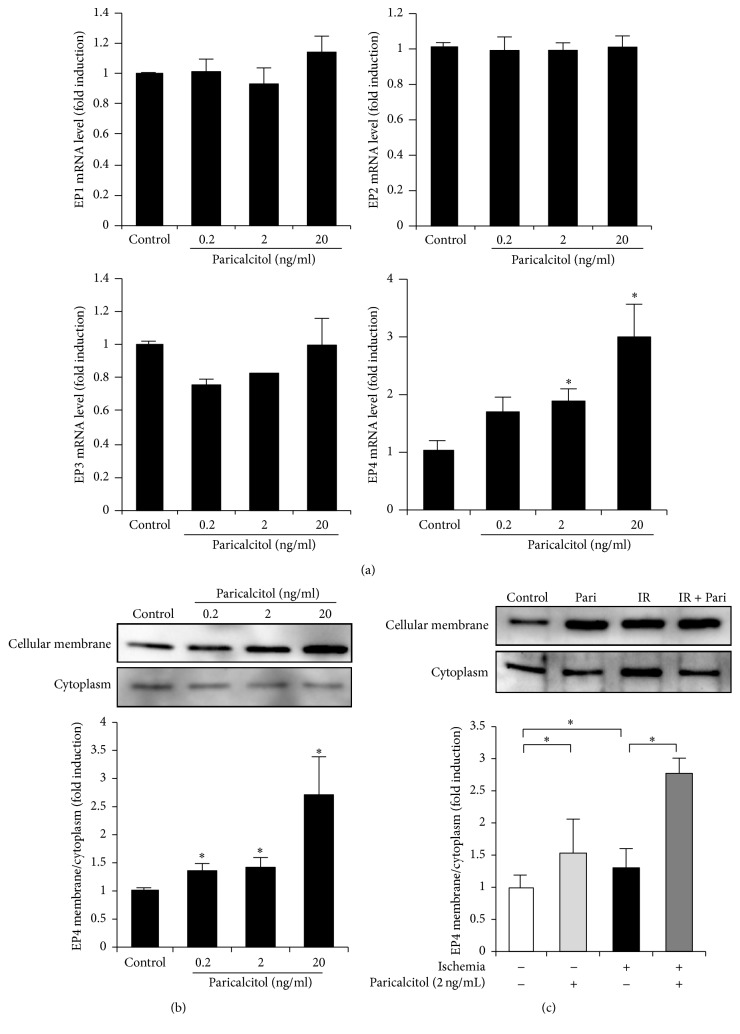
Paricalcitol increased EP4 expression in HK-2 cells. (a) Paricalcitol increased the mRNA level of* EP4* but did not affect mRNA expression of* EP1–EP3*. (b) The cellular membrane fraction of EP4 protein expression was significantly elevated in a dose-dependent manner in response to paricalcitol treatment. (c) The cellular membrane fraction of EP4 protein expression was significantly increased in ischemia-exposed cells and was further increased by treatment with paricalcitol (2 ng/ml). Each column represents the mean ± SEM of three independent experiments. ^*∗*^*P* < 0.05 versus control.

We examined the induction of PGE_2_ receptor subtypes in paricalcitol-treated HK-2 cells. Paricalcitol significantly increased the mRNA levels of* EP4* in a dose-dependent manner, but did not affect mRNA expression of* EP1–EP3* (Figure 2(a)). The cellular membrane fraction of EP4 was increased by paricalcitol treatment in a dose-dependent manner (Figure 2(b)). The cellular membrane/cytoplasm ratio of EP4 was significantly increased in IR-exposed cells compared with the control group and was further enhanced in IR-exposed cells treated with 2 ng/ml paricalcitol (Figure 2(c)).

### 3.3. Paricalcitol Enhances Cell Survival via EP4 in IR-Exposed HK-2 Cells

**Figure 3:**
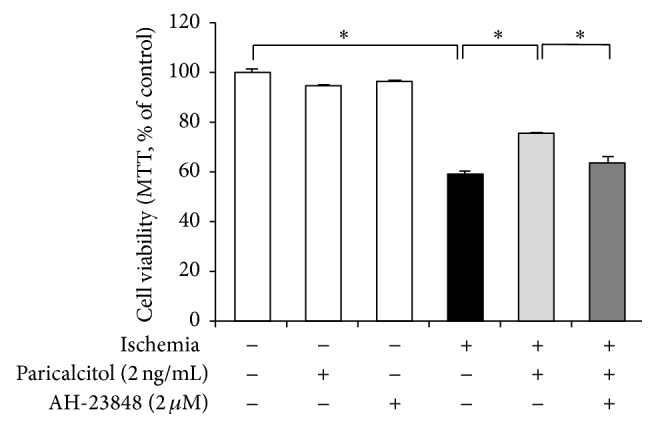
Paricalcitol protected HK-2 cells against ischemia-induced death via an EP4-dependent pathway. Paricalcitol significantly prevented ischemia-induced cell death. Cotreatment with paricalcitol and AH-23848 restored the cell death to such an extent from ischemia-exposed cells without paricalcitol. Each column represents the mean ± SEM of three independent experiments. ^*∗*^*P* < 0.05.

The viability of cells treated with 0.5% NaHCO_3_ was similar to that of control cells without any treatment (*P* = 0.766). IR exposure significantly reduced cell viability to 58.9% of the control group and paricalcitol prevented this IR-induced cell death. This protective effect of paricalcitol in IR-exposed cells was offset by cotreatment of AH-23848 (Figure 3).

### 3.4. Paricalcitol Increases Phosphorylation of Akt and CREB via EP4 in IR-Exposed HK-2 Cells

**Figure 4:**
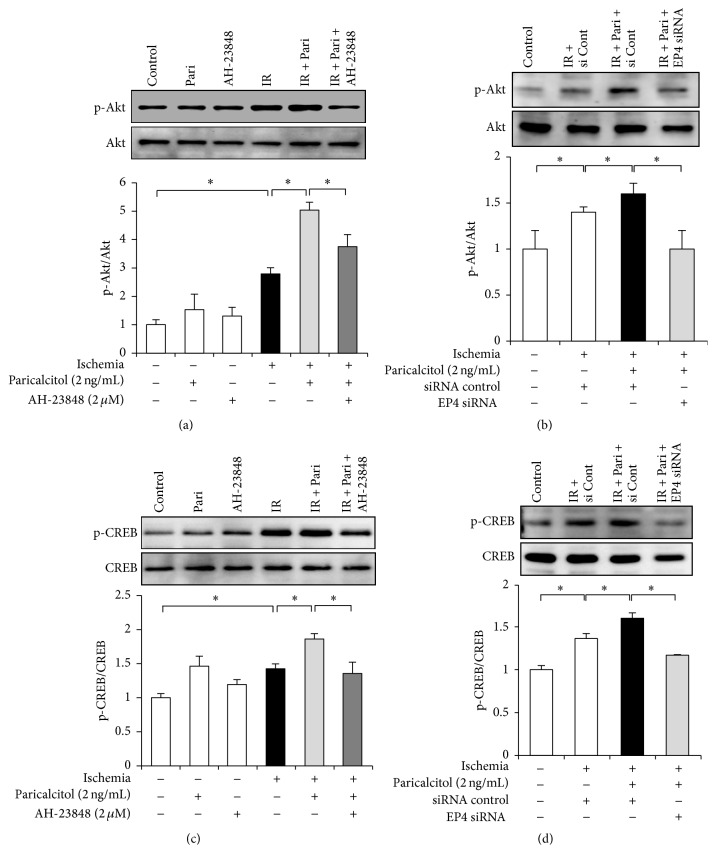
Paricalcitol induced EP4-mediated phosphorylation of Akt and CREB in HK-2 cells with ischemia exposure. (a) Semiquantitative immunoblot analysis showed that in vitro ischemia induced Akt phosphorylation and that it was further enhanced after paricalcitol. The upregulated Akt phosphorylation in paricalcitol-treated cells was attenuated by AH-23848 cotreatment. (b) EP4-specific siRNA significantly inhibited the phosphorylation of Akt in IR-exposed cells treated with paricalcitol. (c) CREB phosphorylation was significantly increased in IR-exposed cells, and paricalcitol pretreatment further increased CREB phosphorylation. AH-23848 cotreatment decreased the phosphorylation of CREB. (d) EP4 siRNA significantly inhibited phosphorylation of CREB in IR-exposed cells treated with paricalcitol. Each column represents the mean ± SEM of three independent experiments. ^*∗*^*P* < 0.05.

Akt phosphorylation was significantly increased in response to IR injury. Paricalcitol further enhanced the phosphorylation of Akt in IR-exposed cells, whereas cotreatment with AH-23848 and paricalcitol decreased phosphorylation of Akt (Figure 4(a)). Similarly, in vitro IR induced phosphorylation of CREB and this was further enhanced by paricalcitol pretreatment. This activation of CREB was reversed by AH-23848 cotreatment (Figure 4(c)). To evaluate whether Akt and CREB were associated with the specific activation of EP4 by paricalcitol treatment, we performed further experiments using EP4 siRNAs in HK-2 cells. EP4 siRNA significantly inhibited the phosphorylation of Akt and CREB in IR-exposed cells treated with paricalcitol (Figures 4(b) and 4(d)).

### 3.5. EP4 Mediates the Inhibitory Effects of Paricalcitol on p65 NF-*κ*B Activation in IR-Exposed HK-2 Cells

**Figure 5:**
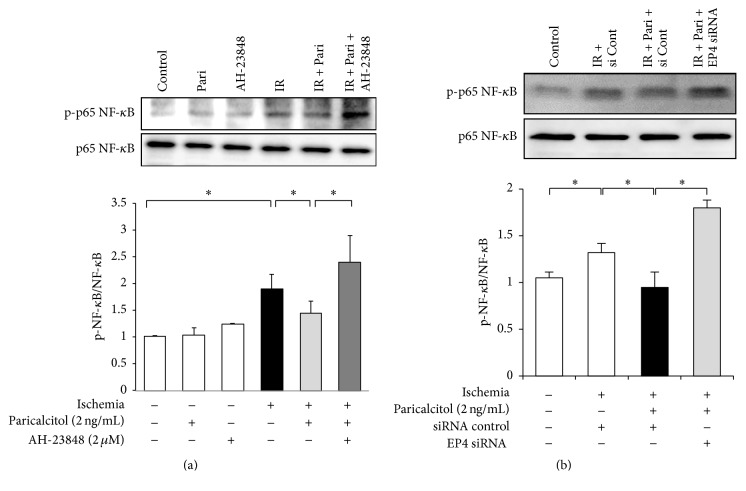
Paricalcitol inhibited p65 NF-*κ*B activation after IR injury via an EP4-dependent pathway in HK-2 cells. (a) Paricalcitol prevented the phosphorylation of p65 NF-*κ*B in IR-exposed HK-2 cells. This inhibitory effect was reversed by AH-23848 cotreatment. (b) IR-exposed cells treated with EP4-specific siRNA and paricalcitol significantly enhanced the phosphorylation of p65 NF-*κ*B compared with IR-exposed cells treated with siRNA control and paricalcitol. Each column represents the mean ± SEM of three independent experiments. ^*∗*^*P* < 0.05.

IR injury increased the phosphorylation of the p65 subunit of NF-*κ*B (Figure 5(a)). Paricalcitol suppressed p65 NF-*κ*B phosphorylation, and this inhibitory effect of paricalcitol was reversed by AH-23848 cotreatment in IR-exposed HK-2 cells. EP4 siRNA also enhanced the phosphorylation of p65 NF-*κ*B in IR-exposed cells treated with paricalcitol (Figure 5(b)).

### 3.6. Paricalcitol Upregulates PGE_2_, Cellular Membrane Expression of EP4, and the Interaction between PGE_2_ and EP4 in Mice Kidney with IR Injury

**Figure 6:**
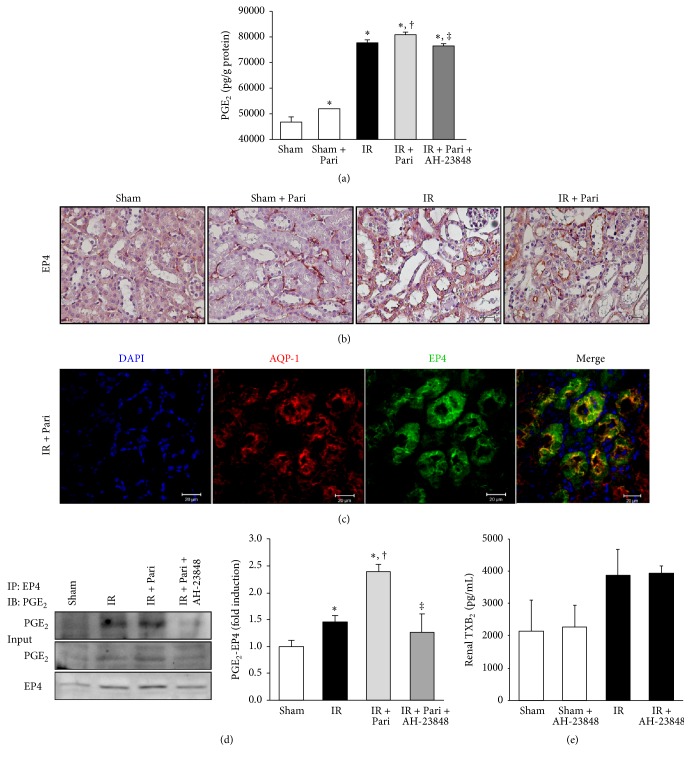
Paricalcitol pretreatment upregulated PGE_2_ and EP4 expression and increased the interaction between PGE_2_ and EP4 in mice kidney with IR injury. (a) PGE_2_ was highly produced after IR injury compared with the sham-operated mice without paricalcitol. Paricalcitol further increased renal PGE_2_ concentrations in mice kidney with IR, but cotreatment with paricalcitol and AH-23848 decreased the PGE_2_ concentrations. (b) The representative staining of EP4 immunoreactivity showed that the cellular membrane expression of EP4 was increased in mice kidney with IR injury and was further enhanced after paricalcitol pretreatment. Original magnification ×400. Bar = 50 *μ*m. (c) The representative image of immunofluorescence staining confirmed that EP4-positive staining was colocalized with the AQP-1 on the cellular membrane of proximal tubule in paricalcitol-treated mice with IR injury. Original magnification ×400. Bar = 20 *μ*m. (d) Immunoprecipitation showed that PGE_2_/EP4 complex formation increased in paricalcitol-treated mice kidney with IR injury compared to mice kidney with IR injury only and significantly decreased in IR injury cotreated with paricalcitol and AH-23848. (e) The concentration of renal TXB_2_ was not significantly different between groups treated with and without AH-23848. ^*∗*^*P* < 0.05 versus sham group, ^†^*P* < 0.05 versus IR group, and ^‡^*P* < 0.05 versus IR + Pari group; *n* = 6–8 for each group.

PGE_2_ levels were highly produced after IR injury compared with the sham-operated mice kidney. Paricalcitol further increased renal PGE_2_ in IR injury, but cotreatment with paricalcitol and AH-23848 decreased PGE_2_ levels (Figure 6(a)). Paricalcitol increased EP4 immunoreactivity on the cellular membrane in sham-operated mice (Figure 6(b)). The cellular membrane expression of EP4 was increased in ischemic mice and further enhanced by paricalcitol pretreatment. We performed immunofluorescence double staining for EP4 and AQP-1, a marker of the proximal tubular brush border. Immunofluorescence analysis confirmed that EP4-positive staining was colocalized with AQP-1 on the cellular membrane of proximal tubule in paricalcitol-treated mice with IR injury (Figure 6(c)).

The interaction between PGE_2_ and EP4 was investigated by immunoprecipitation (Figure 6(d)). PGE_2_/EP4 complex formation was increased after IR injury, and paricalcitol further increased their interaction. The cotreatment with AH-23848 significantly suppressed the complex between PGE_2_ and EP4. The concentration of renal TXB_2_, an inactive product of thromboxane A_2_ (TXA_2_), was measured to exclude involvement of the thromboxane prostanoid pathway in AH-23848-treated mice kidney. There were no significant differences in renal TXB_2_ levels between mice treated with and without AH-23848 (Figure 6(e)).

### 3.7. Paricalcitol Improves Renal Function and Histologic Change via an EP4-Dependent Pathway in Mice Kidney with IR Injury

**Figure 7:**
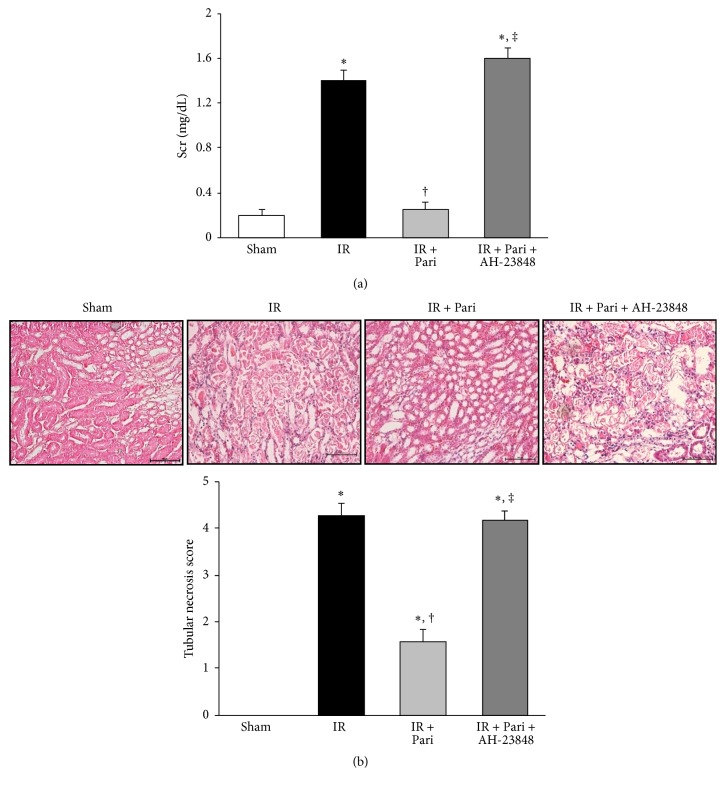
Paricalcitol improved renal function and tubular necrosis via an EP4-dependent pathway in mice kidney with IR injury. (a) Paricalcitol improved serum creatinine levels at day 1 after IR injury, and AH-23848 cotreatment blunted the improvement of serum creatinine level in paricalcitol-treated mice with IR injury. (b) The representative stain of hematoxylin and eosin showed a decreased tubular necrosis in the paricalcitol-treated renal IR injury compared with renal IR injury alone. AH-23848 cotreatment with paricalcitol offset the protective effect on tubular necrosis. Original magnification ×200. ^*∗*^*P* < 0.05 versus sham group, ^†^*P* < 0.05 versus IR group, and ^‡^*P* < 0.05 versus IR + Pari group; *n* = 6–8 for each group. Bar = 50 *μ*m.

Serum creatinine levels were significantly increased at 24 h after IR injury compared with serum creatinine levels in sham-operated mice. Paricalcitol significantly improved serum creatinine levels in ischemic mice. AH-23848 cotreatment blunted the improvement of renal function in paricalcitol-treated ischemic mice (Figure 7(a)). Histologic examination of sections indicated extensive tubular necrosis in the kidneys of ischemic mice compared with those of sham-operated mice (Figure 7(b)). Tubular necrosis was decreased in the paricalcitol-treated mice with IR injury compared with those with IR injury only and AH-23848 cotreatment significantly offset the protective effect of paricalcitol on tubular necrosis.

### 3.8. EP4 Mediates Akt Phosphorylation and p65 NF-*κ*B Nuclear Translocation in Paricalcitol-Treated Mice Kidney with IR Injury

**Figure 8:**
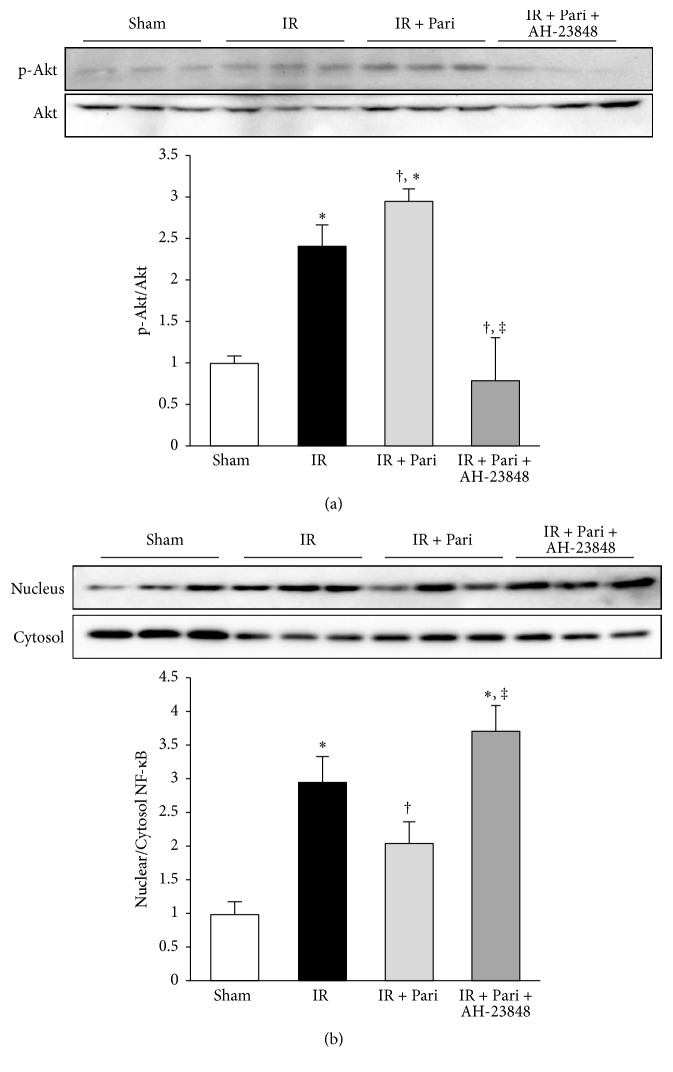
Paricalcitol enhanced Akt phosphorylation and inhibited p65 NF-*κ*B phosphorylation via an EP4-dependent pathway in mice kidney with IR injury. (a) Paricalcitol further enhanced Akt phosphorylation in renal IR injury, but cotreatment with paricalcitol and AH-23848 significantly suppressed this effect. (b) p65 NF-*κ*B phosphorylation was increased in mice kidney with IR injury, and paricalcitol significantly prevented its phosphorylation. This inhibitory effect of paricalcitol was offset by cotreatment with AH-23848. ^*∗*^*P* < 0.05 versus sham group, ^†^*P* < 0.05 versus IR group, and ^‡^*P* < 0.05 versus IR + Pari group; *n* = 6–8 for each group.

Akt phosphorylation was significantly increased in response to IR injury, similar to results of the in vitro study (Figure 8(a)). Paricalcitol further enhanced Akt phosphorylation in mice kidney with IR injury and AH-23848 cotreatment significantly decreased the phosphorylation of Akt. Nuclear translocation of p65 NF-*κ*B was increased in mice kidney with IR injury and significantly suppressed by pretreatment of paricalcitol. This inhibitory effect of paricalcitol was offset by AH-23848 cotreatment (Figure 8(b)).

### 3.9. EP4-Dependent Pathway Contributes to the Antioxidant Effects of Paricalcitol in Mice Kidney with IR Injury

**Figure 9:**
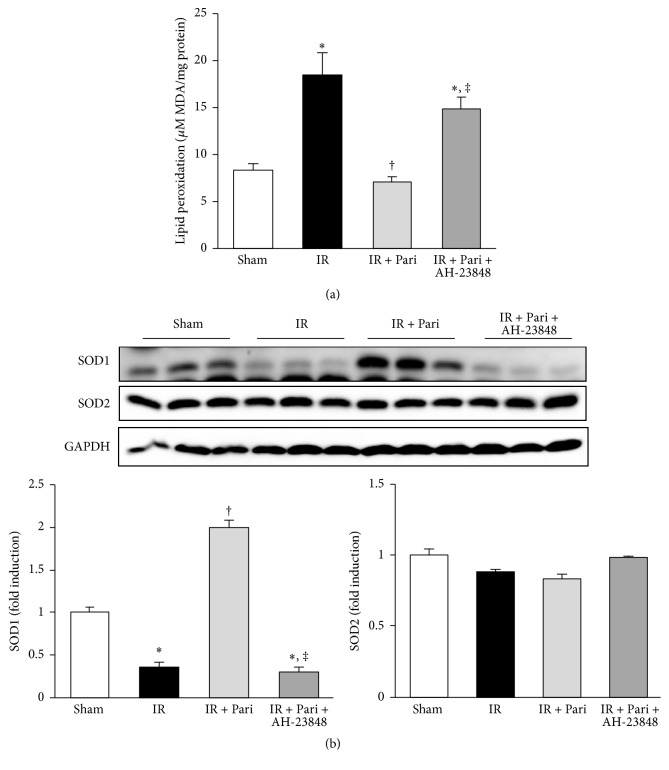
Paricalcitol attenuated oxidative stress via an EP4-dependent pathway in mice kidney with IR injury. (a) IR exposure significantly increased malondialdehyde (MDA) levels and paricalcitol pretreatment significantly alleviated oxidative stress. This protective effect of paricalcitol in renal IR injury was offset by cotreatment of AH-23848. (b) The expression of the superoxide dismutase (SOD) 1 increased in paricalcitol-treated mice kidney with IR injury compared with mice kidney with IR injury alone. This antioxidative effect of paricalcitol was offset by cotreatment with AH-23848. ^*∗*^*P* < 0.05 versus sham group, ^†^*P* < 0.05 versus IR group, and ^‡^*P* < 0.05 versus IR + Pari group; *n* = 6–8 for each group.

Oxidative stress measured by MDA production assay showed that IR exposure significantly increased MDA levels and that paricalcitol pretreatment significantly alleviated oxidative stress. This antioxidant effect of paricalcitol was offset by cotreatment of AH-23848 (Figure 9(a)). IR injury also significantly decreased the expression of SOD1, and paricalcitol recovered SOD1 levels, but not SOD2, in mice kidneys with IR injury. AH-23848 cotreatment decreased SOD1 levels compared to that of paricalcitol-treated mice with IR injury (Figure 9(b)).

### 3.10. EP4-Dependent Pathway Contributes to the Anti-Inflammatory Effects of Paricalcitol in Mice Kidney with IR Injury

**Figure 10:**
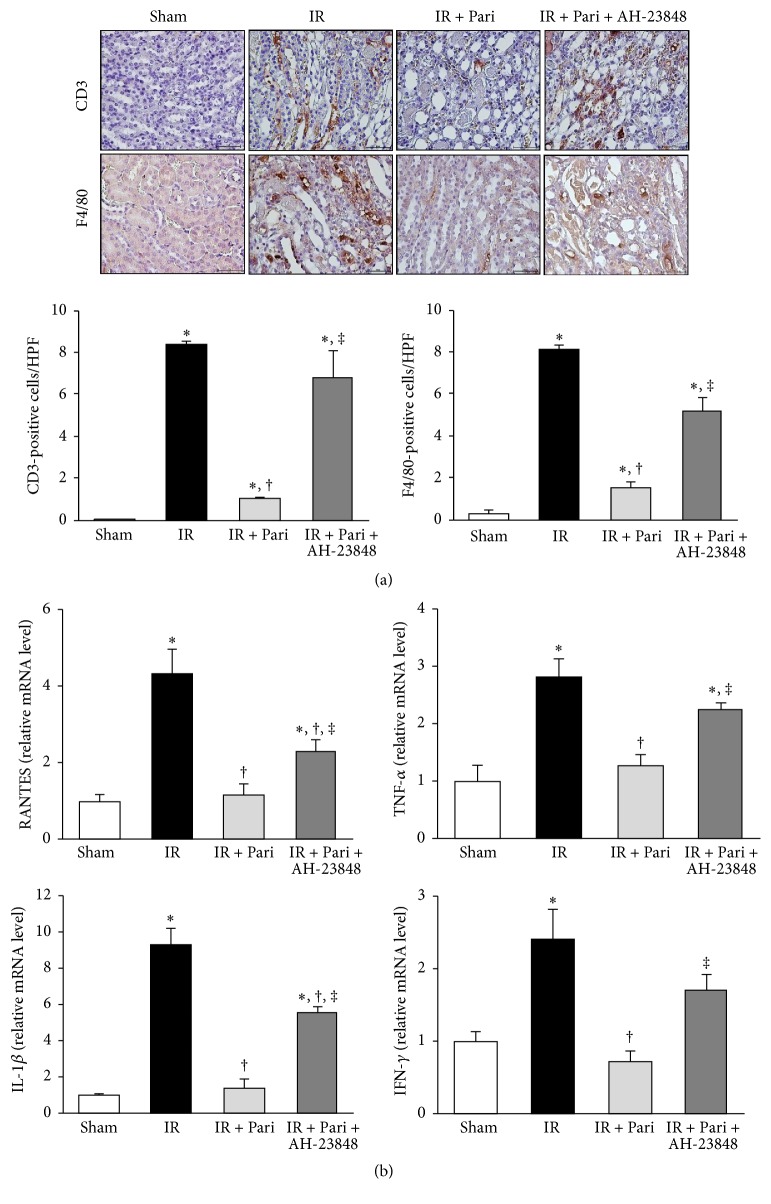
Paricalcitol prevented inflammatory cell infiltration and suppressed the expression of inflammatory cytokines via an EP4-dependent pathway in mice kidney with ischemia-reperfusion (IR) injury. (a) The infiltration of CD3^+^- and F4/80^+^-expressing cells was examined by immunohistochemical staining. The number of CD3^+^- and F4/80^+^-positive cells was increased significantly in mice kidney with IR injury, and this increase was ameliorated by paricalcitol. AH-23848 cotreatment significantly reversed the effect of paricalcitol on inflammatory cell infiltrations into the ischemic kidney. Original magnification ×400. (b) RT-PCR analysis showed that the mRNA levels of* RANTES, TNF-α, IL-1β,* and* IFN-γ*were elevated in mice kidney with IR injury. Paricalcitol suppressed the IR-induced overexpression of these inflammatory cytokines and cotreatment with AH-23848 reversed all of these suppressive effects. ^*∗*^*P* < 0.05 versus sham group, ^†^*P* < 0.05 versus IR group, and ^‡^*P* < 0.05 versus IR + Pari group; *n* = 6–8 for each group. Bar = 50 *μ*m.

IR injury instigated the significant infiltration of CD3^+^ and F4/80^+^ antigen-positive cells in the renal interstitium, but paricalcitol markedly inhibited the infiltration of both types of antigen-positive cells (Figure 10(a)). AH-23848 substantially restored CD3^+^ T-cell and F4/80^+^ monocyte infiltration in paricalcitol-treated mice kidneys with IR injury. We also investigated proinflammatory chemokines that play a crucial role in renal IR injury, such as regulated upon activation normal T-cell expressed and secreted (RANTES), tumor necrosis factor-*α* (TNF-*α*), interleukin-1*β* (IL-1*β*), and interferon-*γ* (IFN-*γ*). Induction of* RANTES, TNF-α, IL-1β, *and* IFN-γ* mRNA was greater in the kidneys with IR injury compared with sham controls (Figure 10(b)). Induction of these proinflammatory cytokines in kidneys with IR injury was significantly reduced by paricalcitol and AH-23848 cotreatment largely reversed this decrease.

### 3.11. EP4-Dependent Pathway Contributes to the Antiapoptotic Effects of Paricalcitol in Mice Kidney with IR Injury

**Figure 11:**
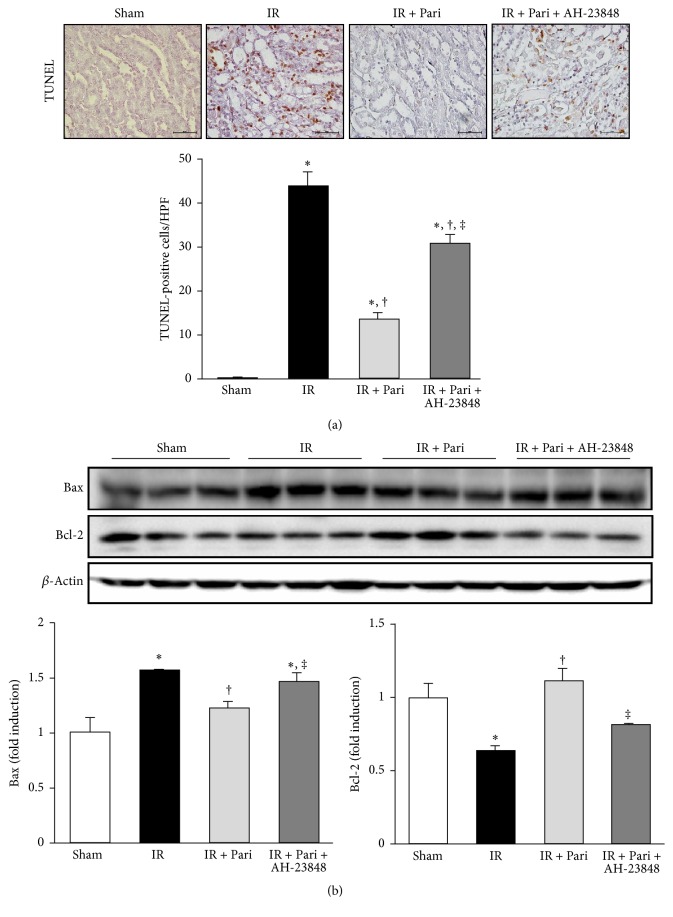
Paricalcitol attenuated apoptosis via an EP4-dependent pathway in mice kidney with IR injury. (a) TUNEL assay revealed that paricalcitol pretreatment significantly reduced the number of TUNEL-positive cells in mice kidney with IR injury, whereas the number of TUNEL-positive cells was increased by AH-23848 cotreatment. Original magnification ×400. (b) Semiquantitative immunoblot analysis indicated that the level of the proapoptotic marker Bax decreased and that of the antiapoptotic marker Bcl-2 increased in paricalcitol-treated mice kidney with IR injury compared with mice kidney with IR injury alone. AH-23848 cotreatment augmented the proapoptotic changes and abolished the antiapoptotic changes. ^*∗*^*P* < 0.05 versus sham group, ^†^*P* < 0.05 versus IR group, and ^‡^*P* < 0.05 versus IR + Pari group; *n* = 6–8 for each group. Bar = 50 *μ*m.

The number of TUNEL-positive cells in mice kidneys with IR injury was significantly attenuated by paricalcitol (Figure 11(a)). However, EP4 blockade significantly increased the number of TUNEL-positive cells in the paricalcitol-treated IR injury. IR injury increased expression of the proapoptotic protein Bax and decreased the expression of the antiapoptotic protein Bcl-2 (Figure 11(b)). Paricalcitol significantly attenuated Bax levels in mice kidneys with IR injury, and AH-23848 cotreatment recovered Bax levels in paricalcitol-treated mice with IR injury. Bcl-2 expression was significantly increased in ischemic mice treated with paricalcitol, whereas AH-23848 cotreatment decreased Bcl-2 levels compared to that of paricalcitol-treated mice with IR injury.

## 4. Discussion

This study demonstrated that paricalcitol pretreatment had renoprotective effects in renal IR injury by upregulating the PGE_2_-EP4 signaling pathway. The paricalcitol-induced EP4 activation mediated phosphorylation of Akt and CREB and inhibited phosphorylation and nuclear translocation of p65 NF-*κ*B in in vitro and in vivo models. As a result, paricalcitol pretreatment had protective effects against cell death in HK-2 cells and against tubular necrosis and apoptosis in mice kidneys with IR injury. In addition, inhibition of renal oxidative stress and inflammation in paricalcitol-treated mice were dependent on PGE_2_-EP4 signaling. These results suggest that paricalcitol improved renal oxidative stress, inflammation, and apoptosis through activation of EP4 and its downstream signaling pathway in ischemic AKI.

Our previous study showed that upregulation of COX-2 is an important mechanism to prevent renal IR injury in paricalcitol-treated mice [12]. In this study, we found that paricalcitol treatment increased the expression of COX-2 and PGE_2_ in HK-2 cells and that it enhanced the PGE_2_ production in ischemic renal tissues. However, the mRNA level of* 15-PGDH* and* PGES*, which are also involved in the degradation and synthesis of PGE_2_ was not affected by paricalcitol. These findings indicate that paricalcitol increased PGE_2_ production primarily through COX-2 rather than 15-PGDH or PGES.

PGE_2_ elicits its biological effects through EP1 to EP4 and our study demonstrated that paricalcitol specifically increased the expression of EP4, not EP1–EP3. Paricalcitol pretreatment also increased the cellular membrane expression of EP4 in both in vitro and in vivo proximal tubular cells, which implies that EP4 signal transduction cascades are initiated [22]. In addition, EP4 expression in IR-exposed cells and ischemic mice kidney was further increased by paricalcitol pretreatment and the interaction between PGE_2_ and EP4 was strengthened after paricalcitol treatment. These findings suggest that paricalcitol is a crucial activator of PGE_2_-EP4 signaling pathway in the kidney with IR injury.

We investigated the effect of EP4 blockade to elucidate the EP4-specific pathway underlying the activity of paricalcitol. EP4 siRNA and cotreatment with an EP4 antagonist significantly offset the protective effects of paricalcitol in IR-induced cell death. In mice kidney with IR injury, EP4 blockade also offsets the improvement of renal function and tubular necrosis. In addition, EP4 siRNA and AH-23848 reversed Akt and CREB phosphorylation and NF-*κ*B inactivation, which are activated downstream signals of the EP4 pathway. Because AH-23848 has a partial antagonistic effect on TXA_2_ receptor, we measured the levels of renal TXB_2_, an inactive product of TXA_2_ [23, 24]. The measured TXB_2_ levels were not different between mice kidney treated with and without AH-23848. Therefore, our data on EP4 blockade suggest that EP4-specific pathway is a critical mechanism in the renoprotective effect of paricalcitol in IR injury.

The action of EP4 is traditionally considered to depend on the activation of protein kinase A, which phosphorylates downstream effector proteins, in particular CREB. PGE_2_ signaling through the EP4 receptor also activates PI3K/Akt signaling pathways [14]. Phosphorylation of Akt and CREB activates numerous downstream kinases to cause antiapoptotic and cell survival effects [25–27]. In the present study, PGE_2_-EP4 signaling mediated paricalcitol-induced phosphorylation of Akt and CREB in both in vitro and in vivo IR models. In addition, EP4 was responsible for decreasing the number of TUNEL-positive cells, decreasing Bax levels, and increasing Bcl-2 expression in ischemic mice kidney pretreated with paricalcitol. These findings suggest that EP4 promotes the antiapoptotic and survival effects of paricalcitol in renal IR injury through activation of Akt and CREB.

The activation of PI3K/Akt signaling pathways and CREB-driven transcription are major protective mechanisms against oxidative stress [25, 28]. Our study demonstrated that EP4 mediated the phosphorylation of Akt and CREB in both in vitro and in vivo IR injury and that EP4 activation also determined the paricalcitol-induced suppression of NF-*κ*B, which leads to the generation of reactive oxygen species [29, 30]. Paricalcitol also decreased MDA levels and increased SOD1 expression of renal tissue in EP4-dependent manner. In addition, our supplementary experiment revealed that paricalcitol prevented H_2_O_2_-induced cell death through the EP4 pathway (see Supplement Figure 1 in Supplementary Material available online at https://doi.org/10.1155/2017/5031926). Therefore, our data suggest that EP4-dependent pathway is important in antioxidant effects of paricalcitol in renal IR injury.

Tubular epithelial cells are the largest cell population involved in the development of renal inflammation, and NF-*κ*B is a key transcription factor initiating the inflammatory cascade [31, 32]. Although the inhibitory effects of paricalcitol on NF-*κ*B phosphorylation have previously been reported, there are no reports that have supported the EP4-mediated NF-*κ*B regulation in paricalcitol treatment [18, 33]. Our data indicated that EP4 controlled the inhibitory effect of paricalcitol on NF-*κ*B activation in both in vitro and in vivo models. We also demonstrated that paricalcitol suppressed the expression of proinflammatory cytokines and prevented the infiltration of T-cells and macrophages through an EP4-dependent pathway. It is well known that NF-*κ*B signaling provokes the expression of RANTES, and TNF-*α* augments this response in tubular epithelial cells. Furthermore, a number of studies have reported that EP4 agonists attenuate the release of IL-1*β* from macrophages and suppress the T-cells to produce IFN-*γ* [16, 34, 35]. Therefore, we suggest that paricalcitol exerts anti-inflammatory effect in renal IR injury via activation of the EP4 pathway.

Recently, Ranganathan et al. demonstrated that administration of EP4 agonist exacerbates renal IR injury and abolished netrin-1-mediated suppression of neutrophil infiltration [36]. These results seem contradictory to our results. However, in other animal model, a ureteral obstruction model, the development of renal tubulointerstitial fibrosis and the formation of proinflammatory cytokines were significantly augmented in the* EP4 *knock-out mice kidney. Moreover, an EP4-specific agonist attenuated renal injury in animal model of folic acid administration and ureteral obstruction [37]. These findings suggest that the role of EP4 activation might differ depending on various conditions of kidney injury. Therefore, we suggest that EP4 activation plays a protective role in the experimental setting of paricalcitol pretreatment for renal IR injury.

Vitamin D deficiency has an important role as a risk factor and predictor of clinical outcomes of AKI in critically ill patients. Paricalcitol has been widely used in various clinical settings with few adverse events and good tolerance; our study indicates that paricalcitol has antiapoptotic, antioxidative, and anti-inflammatory effects via PGE_2_-EP4 signaling pathway in renal IR injury model. Therefore, we suggest that paricalcitol may have significant potential in therapeutic intervention for ischemic AKI via EP4-dependent mechanism.

In conclusion, our study demonstrated that paricalcitol upregulated PGE_2_-EP4 signaling, and subsequent activation of the EP4 pathway mediated activation of Akt and CREB and inhibition of NF-*κ*B activation. These actions of paricalcitol on PGE_2_-EP4 signaling pathway contributed to its antioxidant, anti-inflammatory, and antiapoptotic properties in renal IR injury. Therefore, the PGE_2_ receptor EP4 plays a pivotal role in the protective effects of paricalcitol in renal IR injury.

## Supplementary Material

Supplement Figure 1: Paricalcitol protected HK-2 cells against H_2_O_2_-induced death via an EP4-dependent pathway. Paricalcitol significantly prevented H_2_O_2_-induced cell death. Co-treatment with paricalcitol and AH-23848 restored the cell death to such an extent from H_2_O_2_-exposed cells without paricalcitol. Each column represents the mean ± SEM of three independent experiments. ^*^P < 0.05.

## Figures and Tables

**Table 1:** Primer sequences for real-time PCR.

Gene	Sequence
*mRANTES*	Fwd: CACCACTCCCTGCTGCTTTG
	Rev: CGACTGCAAGATTGGAGCACTT
*mTNF-α*	Fwd: ACGGCATGGATCTCAAAGAC
	Rev: GTGGGTGAGGAGCACGTAGT
*mIL-1β*	Fwd: GCCCATCCTCTGTGACTCAT
	Rev: AGGCCACAGGTATTTTGTCG
*mIFN-γ*	Fwd: GCTACACACTGCATCTTGGCTTT
	Rev: AATGACTGTGCCGTGGCAGTAA
*hCOX-2*	Fwd: GATACTCAGGCAGAGATGATCTACCC
	Rev: AGACCAGGCACCAGACCAAAGA
*hPGES*	Fwd: CCTGGGCTTCGTCTACTCCTTT
	Rev: CCCACGAGGAAGACCAGGAA
*h15-PGDH*	Fwd: GAAGGCGGCATCATTATCAATATG
	Rev: GCTGAGCGTGTGAATCCAACTAT
*hEP1*	Fwd: TTGTCGGTATCATGGTGGTG
	Rev: ATGTACACCCAAGGGTCCAG
*hEP2*	Fwd: CCTGCTGCTGCTTCTCATTGTCT
	Rev: GGCTTCGGCGGTGCAT
*hEP3*	Fwd: AAGGCCACGGCATCTCAGT
	Rev: ACGCACATGATCCCCATAAGC
*hEP4*	Fwd: TCCGGCCTCAGCATCATC
	Rev: GGCTGTAGAAATAGGCATGGTTGA
*mGAPDH*	Fwd: TGCAGTGGCAAAGTGGAGATT
	Rev: CGTGAGTGGAGTCATACTGGAACA
*hGAPDH*	Fwd: GAAAAACCTGCCAAATATGATGACA
	Rev: TAGCCCAGGATGCCCTTGAG

m, mouse; h, human; Fwd, forward; Rev, reverse; RANTES, regulated on activation normal T-cell expressed and secreted; TNF-*α*, tumor necrosis factor-*α*; IL-1*β*, interleukin-1*β*; IFN-*γ*, interferon-*γ*; COX, cyclooxygenase; PGES, prostaglandin E synthases; 15-PGDH, 15-hydroxyprostaglandin dehydrogenase; EP, prostaglandin receptor; GAPDH, glyceraldehyde-3-phosphate dehydrogenase.

## Abbreviations

15-PGDH: 15-Hydroxyprostaglandin dehydrogenase
AKI: Acute kidney injury
AQP-1: Aquaporin-1
Bax: BCL-2-associated X
Bcl-2: B-cell leukemia/lymphoma 2
COX-2: Cyclooxygenase-2
CREB: Cyclic AMP responsive element binding protein
GAPDH: Glyceraldehyde-3-phosphate dehydrogenase
HK-2: Human proximal tubular epithelial cells
IFN-*γ*: Interferon-*γ*
IL-1*β*: Interleukin-1*β*
IR: Ischemia-reperfusion
NF-*κ*B: Nuclear factor-*κ*B
PGE_2_: Prostaglandin E_2_
PGES: Prostaglandin E synthases
PI3K: Phosphatidylinositol 3-kinase
RANTES: Regulated upon activation normal T-cell expressed and secreted
ROS: Reactive oxygen species
siRNA: Small interfering RNA
SOD: Superoxide dismutase
TNF-*α*: Tumor necrosis factor-*α*
TUNEL: Terminal deoxynucleotidyl transferase-mediated dUTP nick end-labeling
TXA_2_: Thromboxane A_2_
TXB_2_: Thromboxane B_2_
VDR: Vitamin D receptor.
