# Corrigendum to “Paricalcitol Pretreatment Attenuates Renal Ischemia-Reperfusion Injury via Prostaglandin E_2_ Receptor EP4 Pathway”

**DOI:** 10.1155/2017/1913954

**Published:** 2017-08-13

**Authors:** Yu Ah Hong, Keum Jin Yang, So Young Jung, Ki Cheol Park, Hyunsu Choi, Jeong Min Oh, Sang Ju Lee, Yoon Kyung Chang, Cheol WheePark, Chul Woo Yang, Suk Young Kim, Hyeon Seok Hwang

**Affiliations:** ^1^Division of Nephrology, Department of Internal Medicine, College of Medicine, the Catholic University of Korea, Seoul, Republic of Korea; ^2^Clinical Research Institute, Daejeon St. Mary's Hospital, Daejeon, Republic of Korea

In the article titled “Paricalcitol Pretreatment Attenuates Renal Ischemia-Reperfusion Injury via Prostaglandin E_2_ Receptor EP4 Pathway” [1], an acknowledgement should be added as follows:

The authors also wish to acknowledge the financial support of the Korean Society of Nephrology (JW Pharmaceutical, 2013).
