# Supplementary figures and images for: Paricalcitol Pretreatment Attenuates Renal Ischemia-Reperfusion Injury via Prostaglandin E2 Receptor EP4 Pathway
Source: Oxid Med Cell Longev. 2017 Mar 29;2017:5031926. doi: 10.1155/2017/5031926 (PMC5390586; doi:10.1155/2017/5031926)

Supplement figure 1.

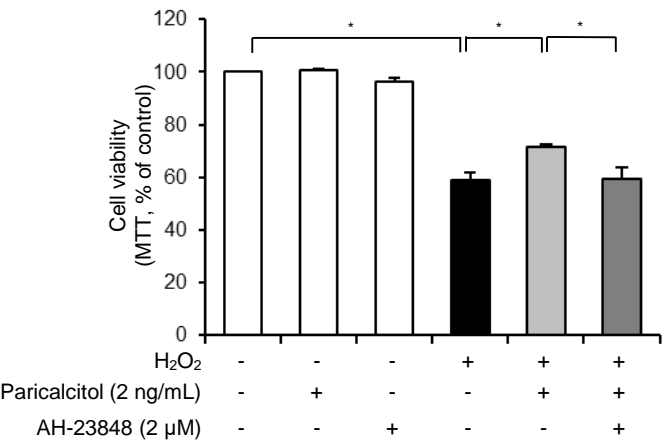

Supplement: Supplementary file 1 — Supplement Figure 1: Paricalcitol protected HK-2 cells against H2O2-induced death via an EP4-dependent pathway. Paricalcitol significantly prevented H2O2-induced cell death. Co-treatment with paricalcitol and AH-23848 restored the cell death to such an extent from H2O2-exposed cells without paricalcitol. Each column represents the mean ± SEM of three independent experiments. *P < 0.05. [file 5031926.f1.pdf]
